# Identification and Characterization of Dimorphic Expression of Sex-Related Genes in Rock Bream, a Fish With Multiple Sex Chromosomes

**DOI:** 10.3389/fgene.2021.791179

**Published:** 2021-11-29

**Authors:** Huan Li, Qihui Zhu, Ruiyi Chen, Mingtao Liu, Dongdong Xu

**Affiliations:** ^1^ School of Fisheries, Zhejiang Ocean University, Zhoushan, China; ^2^ Key Lab of Mariculture and Enhancement of Zhejiang Province, Zhejiang Marine Fisheries Research Institute, Zhoushan, China; ^3^ Ocean and Fisheries Research Institute, Zhejiang Ocean University, Zhoushan, China

**Keywords:** gonadal development, RNA-seq, histological observation, rock bream, sex-related genes

## Abstract

The rock bream (*Oplegnathus fasciatus*) is a typical fish with a unique multiple sex chromosome system. In this study, we investigated the gene expression profiling in the gonads and brains of both males and females using RNA-Seq to identify sex-related genes and pathways. In accordance with the dimorphic expression profiles, combined with Gene ontology and KEGG enrichment analyses, a number of potential genes and pathways associated with sex determination were obtained from transcriptional analysis, especially some sex-biased genes and pathways. Next, we selected 18 candidate genes and analyzed their expression in different tissues and developmental stages. We found that the expression levels of *Amh*, *Dmrt1*, *Sox9*, *Dmrtb1*, and *Nanos2* were significantly higher in the testis than those in the ovary or other tissues, whereas the expression levels of *ZP4*, *Bouncer*, *RNF208*, *FoxH1*, and *TOB* were significantly higher in the ovary than those in the testis. Furthermore, the expression levels of these genes in different developmental stages of gonads also showed sexually dimorphic patterns, suggesting that they might play important roles during gonadal development. These genes are useful markers for investigating sex determination and differentiation in rock bream. The findings of this study can provide insights into the molecular mechanisms of sex determination and differentiation in fish with multiple sex chromosome systems.

## Introduction

Teleost fish display an amazing diversity of sex-determination systems, such as XX/XY, ZZ/ZW, XX/XO, and ZZ/ZO. Systems that are more complicated can involve multiple sex chromosomes and multiple gene loci (influence of autosomal loci on sex determination and polyfactorial sex determination). The multiple ♀X_1_X_1_X_2_X_2_/♂X_1_X_2_Y sex chromosome system is characterized by a neo-Y sex chromosome, with males containing an exceptionally large heterotypic chromosome and possessing one less chromosome than females. To date, over 50 cases with multiple sex chromosomes have been reported in teleost phylogeny (Sember et al., 2015; Bitencourt et al., 2017; Zhang et al., 2018a; Krysanov and Demidova, 2018; Cai et al., 2019; Xu et al., 2019). However, little is known about the molecular sex determination mechanism of multiple sex chromosome system in fish. The rock bream (*Oplegnathus fasciatus*), a member of the Oplegnathidae family of the Centrarchiformes, is a commercially important rocky reef fish native to East Asia and has been reported as a typical fish with multiple X_1_X_1_X_2_X_2_/X_1_X_2_Y sex chromosome system (Xu et al., 2012; Xue et al., 2016; Xu et al., 2019). Owing to their high aquaculture value, unique sex-determining system, and susceptibility to widespread biotic diseases, rock bream fish have drawn increasing research interest. For example, studies have been conducted on the characterization of the differences between X and Y chromosomes by molecular markers and cytological genetic methods and preliminary clarification of the origin and differentiation of a multiple X_1_X_2_Y sex chromosome system (Xu et al., 2013a; Xu et al., 2013b; Xu et al., 2019). Moreover, the whole genomes of both male and female rock breams were sequenced, assembled, and analyzed, and their results suggested that the neo-Y chromosome probably originated from the centric fusion of acrocentric chromosomes (X_1_ and X_2_ chromosomes) (Shin et al., 2018; Xiao et al., 2019; Xiao et al., 2020). These studies have contributed to the understanding of sex determination in species with a unique multiple sex chromosome system. Nevertheless, to date, little is known about the molecular pathways and regulatory mechanisms of sex determination in fish with multiple sex chromosome system.

Genetic sexual determination has always been a hot topic in the field of aquatic research and involves a series of complex biochemical interactions that lead to gonadal sexual determination and differentiation. Genetic systems in vertebrates may operate by polygenic control mechanisms and dominant genes, along with autosomic controls and sex chromosomes. The genes involved in sexual determination may be distributed in the genome, localized in individual chromosomes, or restricted to a single genetic locus (Robert and Nagahama, 2002). In monogenic systems, sex is determined by a specific gene on the sex chromosome, which has strong specificity and functionality. Only a handful of sex-determining genes have been identified in fish, including the Y chromosome-linked anti-Müllerian hormone (*Amhy*) gene of *Odontesthes hatcheri* (Ricardo et al., 2012) and Nile tilapia (*Oreochromis niloticus*) (Sissao et al., 2019), *Sdy* of rainbow trout (*Oncorhynchus mykiss*) (Yano et al., 2012), and anti-Müllerian hormone type II receptor (*AmhrII*) of Fugu rubripes (*Takifugu rubripes*) (Kamiya et al., 2012). A number of genes were identified to be closely related to sexual determination and differentiation in the complex sexual determination system in fish. For example, the anti-Müllerian hormone (*Amh*), which is expressed in Sertoli cells, has been reported to inhibit the expression of ovary specific genes in Nile tilapia (*O. niloticus*) (Ijiri et al., 2008). Although studies on Nile tilapia (*O. niloticus*) have shown that *Amh* plays a key role in male sex determination, the regulatory mechanism of this gene remains unclear. A number of genes related to sexual determination and differentiation have been discovered in many fishes, such as *Nanos2* (Tsuda et al., 2003), *Sox9* (Chen et al., 2015), and *Gsdf* (Jiang et al., 2016). These studies have provided a comprehensive understanding of sex determination in fish, whereas information on the mechanism of sexual determination in the multiple sex chromosome system is still scarce.

RNA-Seq has been proven to be a powerful strategy for quantifying gene expression levels, even when the expression levels are low. Transcriptomic studies on gonads have been conducted in a relatively large number of fish species, including Nile tilapia (*O. niloticus*) (Tao et al., 2013; Tao et al., 2018), spotted knifejaw (*Oplegnathus punctatus*) (Li et al., 2020), fugu rubripes (*T. rubripes*) (Yan et al., 2018), and spotted scat (*Scatophagus argus*) (He et al., 2019). For example, in the channel catfish (*Ictalurus punctatus*), male-preferential genes, such as *Gsdf* and *cxcl12*, were identified by transcriptomic comparison of the testes and ovaries (Zeng et al., 2016). In the spotted knifejaw (*O. punctatus)*, a set of unigenes related to gonadal development and gametogenesis was filtered by transcriptomic analysis and genes, such as *bmp15*, *Nanos3*, *Sox9*, and *Amh*, were found to have roles in the regulation of gonadal physiology and germ line cell maintenance in the ovaries and testes (Du et al., 2017).

In this study, transcriptional analyses were carried out on rock bream to investigate the male and female gonads at different developmental stages to filter potential genes and pathways associated with sex determination. Next, several genes were screened and further analyzed by quantitative PCR. The results of this study will increase our understanding of the molecular mechanisms involved in sex determination and differentiation in fish with multiple sex chromosomes.

## Materials and Methods

### Ethics Statement

The collection and handling of all animals used in this study were approved by the Animal Care and Use Committee of Zhejiang Ocean University and the Zhejiang Marine Fisheries Research Institute. All experimental procedures were performed in accordance with the prescribed guidelines.

### Samples and Tissue Collection

The experimental fish were reared in the hatchery of the Zhejiang Marine Fisheries Research Institute, Xixuan Island, Zhoushan, China. The rock breams were randomly sampled at the age of 1 (245.50 ± 16.82 g), 1.5 (315.45 ± 18.5 g) and 2Y (year old) (397.68 ± 20.05 g). At each sampling time point, 10 fish were randomly chosen and dissected immediately. The brain samples were immediately stored in liquid nitrogen for RNA isolation and each gonad sample was divided into two aliquots: one was stored in liquid nitrogen and the other was fixed with Bouin’s solution for histological analysis. The brain and gonad tissues from the 1.5Y rock bream were also prepared for transcriptional analysis via RNA-seq. For the 1.5Y rock breams, in addition to the brain, gonad, heart, liver, kidney, gill, intestine, and muscle were collected and stored in liquid nitrogen. After sex detection by histological observation of the gonad tissue, three samples from each male and female from each sampling time were randomly chosen for RNA isolation and subsequent analyses.

### Total RNA Extraction and Sequencing

Samples of the gonads, brain, and other tissues were stored in liquid nitrogen for gene expression analysis. Total RNA was extracted using a Takara RNA kit (RR047A, Dalian), and the quality was determined using 1.2% denatured agarose gel. The concentration and quality of RNA were detected by AB-Sorbance and agarose gel electrophoresis at 260 nm, respectively. With samples for transcriptional analysis (three samples of each gonad and brain tissues from both male and female fish), RNA integrity was assessed using an Agilent 2100 biological analyzer (Agilent technologies), and samples with RNA integrity numbers ≥7 were subsequently analyzed. Libraries were constructed and sequenced using the BGISEQ-500 platform.

### Transcriptional Analysis and Functional Annotation

Raw data were processed using the SOAPnuke method. After removing the adaptor and low-quality sequences, the clean reads were subsequently aligned with Bowtie2 and mapped to the rock bream genome (NCBI accession number SRP220007) using HISAT. The FPKM (Fragments Per Kilobase of exon model per Million mapped fragments) (Trapnell et al., 2010) and read count values of each gene were calculated using Bowtie2 (Langmead and Salzberg, 2012) and RSEM (Li and Dewey, 2011). Pearson correlation coefficients between two samples were calculated using the core function in R software. The DEGseq method was based on the Poisson distribution, and the present project performed differentially expressed gene (DEG) detection according to a previously described method (Anders and Huber, 2012). To improve the accuracy of DEGs, we defined the genes with more than two-fold difference and Q-value ≤ 0.001 (fold change ≥ 2 and adjusted *p*-value ≤ 0.001), which were screened as significant DEGs. Gene ontology (GO) enrichment and Kyoto Encyclopedia of Genes and Genomes (KEGG) pathway enrichment analyses of DEGs were performed in R based on hypergeometric distribution.

### Quantitative Polymerase Chain Reaction (qPCR) Validation

Quantitative PCR was performed on a StepOnePlus Real-Time PCR system (Thermo Fisher Scientific, United States) using SYBR Green Real-time PCR Mix (RR420A; Takara, Dalian), following the manufacturer’s protocol. An isolation step was conducted after each PCR run to verify amplification specificity. Three fish samples were randomly chosen as biological replicates in qPCR. For each sample, qPCR was performed in triplicate. Primer pairs for qPCR were designed using the Primer Premier V6.0. The details of these genes are listed in Supplementary Table S1. Target gene expression was analyzed using the 2^−ΔΔCT^ method (Kenneth and Thomas, 2001). The copy number of a reference gene, *β-actin*, was used to normalize the expression values.

### Histology Observation

The gonads were dissected and fixed in the Bouin’s solution, and the samples were embedded in paraffin using conventional techniques. Sections (5 μm) were cut, mounted on slides, and stained with hematoxylin and eosin. Images of the sections were obtained using a microscope and digital camera (Axio Imager A2; digital camera (Axiocam 506; Zeiss). The phenotypic sex of each fish was determined, and the development of testes and ovaries was observed.

## Results

### Transcriptome Data Analysis

A total of 12 samples were analyzed via RNA-Seq in this study, with three samples in each group, including ovary (F_sex group), testis (M_sex group), female brain (F_brain group), and male brain (M_brain group). As a result, an average output of 6.50 GB bases per sample was generated, with an average clean reads ratio of up to 89.93%. Detailed statistics of the transcriptome data are summarized in Supplementary Table S2. As the average genome mapping rate of samples was 90.96%, a total of 25,678 genes were obtained, of which 22,723 were known genes and 2,955 were predicted new genes. A total of 41,127 new transcripts were obtained, of which 29,536 were new alternative splicing subtypes of known protein-coding genes, 2,974 were new protein-coding genes, and the remaining 8,617 were long non-coding RNAs. The correlation coefficients between replicated samples were higher than 0.88 (Supplementary Figure S1), indicating the conserved similarity between the replicates, and the results also showed good correlation coefficients between all brain samples. The results of principal component analysis showed that the replicates of ovaries, testes, and brains were clustered closely in a region, further validating the results of the correlation coefficient analysis (Supplementary Figure S2). DEGs between males and females were identified (Supplementary Figure S3). The F_sex-vs-M_sex group contained 17,033 upregulated and 2024 downregulated DEGs, whereas the F_brain-vs-M_brain group contained only 1,325 upregulated and 523 downregulated DEGs.

**FIGURE 1:**
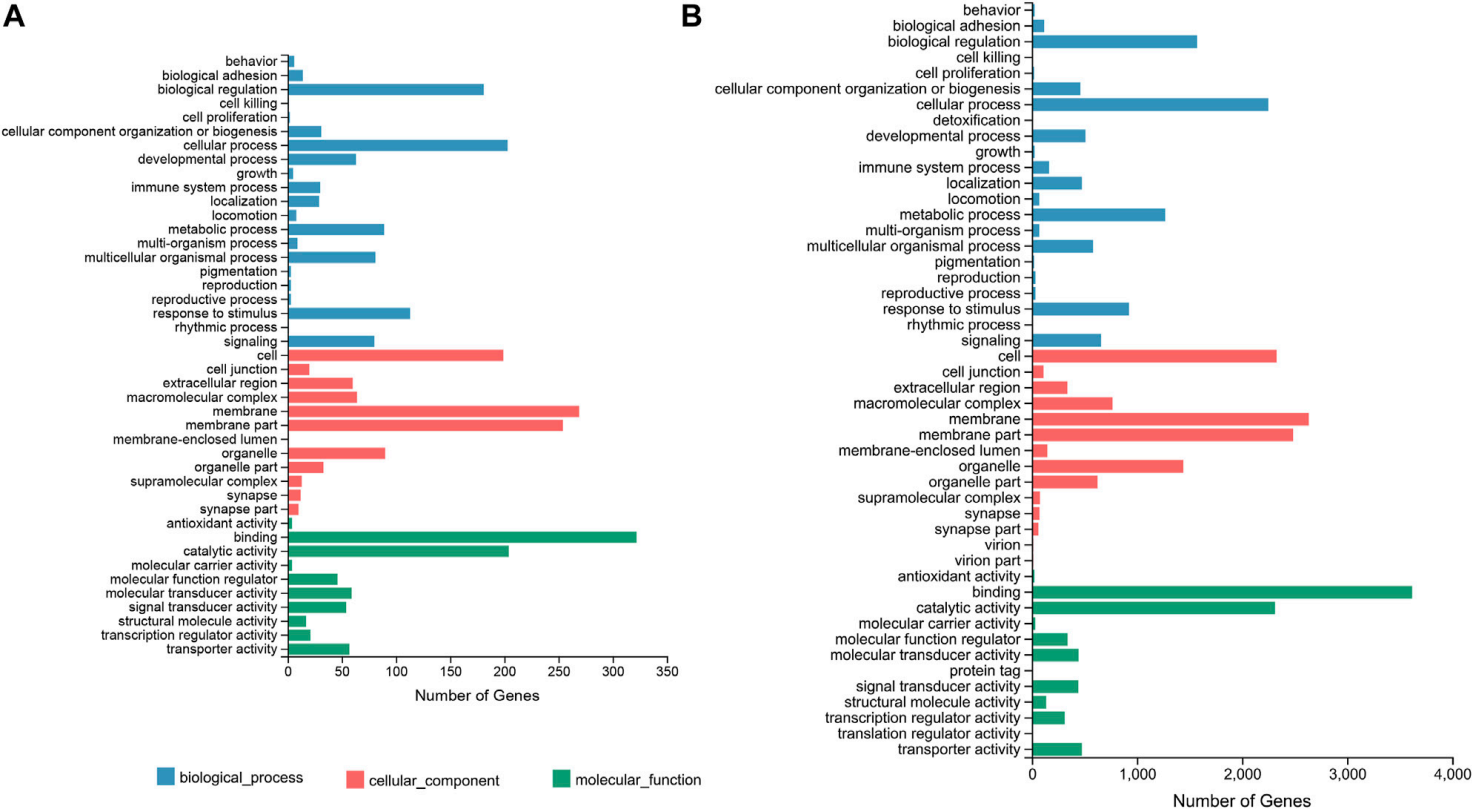
GO classification based on the DEGs of the F_brain-vs-M_brain group **(A)** and the F_sex-vs-M_sex group **(B)**. The horizontal axis shows the number of DEGs enriched in each GO term. GO, gene ontology; DEG, differentially expressed gene.

**FIGURE 2:**
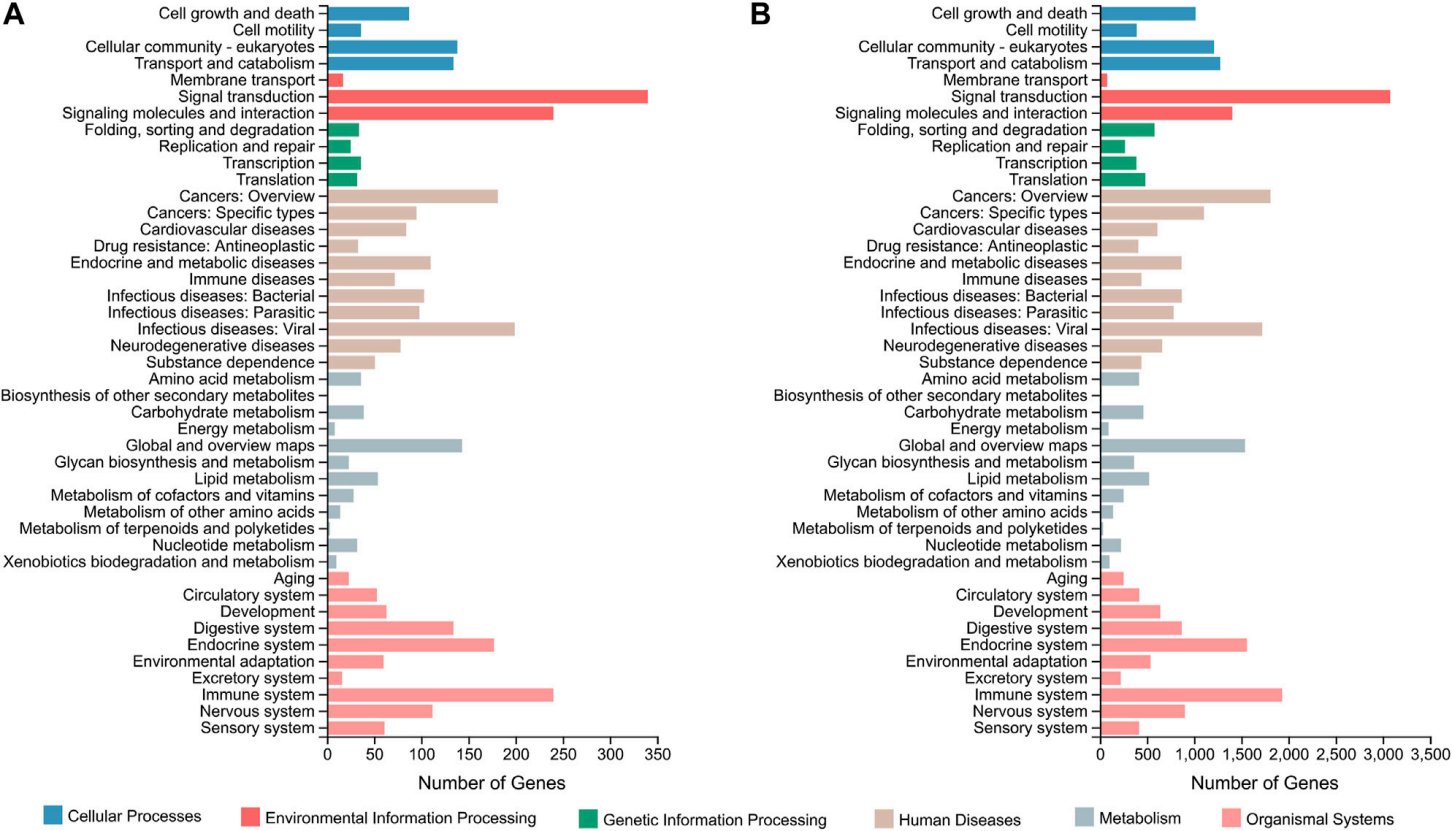
KEGG classification based on the DEGs of the F_brain-vs-M_brain group **(A)** and the F_sex-vs-M_sex group **(B)**. The horizontal axis shows the number of DEGs enriched in each KEGG term. KEGG, Kyoto Encyclopedia of Genes and Genomes; DEG, differentially expressed gene.

In terms of the DEGs of F_brain-vs-M_brain group, GO classification results (Figure 1A) highlighted that GO terms of “biological regulation” (181 genes) and “cellular process” (203 genes) in the biological process category, “membrane” (269 genes) and “membrane part” (254 genes) in the cellular component category, and “catalytic activity” (204 genes) and “binding” (322 genes) in the molecular function category contained the highest number of DEGs. Further GO enrichment analysis showed that a total of 50 GO terms were significantly enriched (Q value < 0.05), including several GO terms relating to development or differentiation, such as “central nervous system development” and “spinal cord motor neuron differentiation” (Supplementary Figure S4). KEGG annotation results showed that for the DEGs between male and female brains, the terms of “signal transduction” (340 genes), and “signaling molecules and interaction” (240 genes), were classified with the highest number of genes (Figure 2A). KEGG enrichment analysis showed that 29 pathways were significantly enriched (Q value < 0.05), of which 10 belonged to the category of “organismal systems,” and the pathway of “neuroactive ligand-receptor interaction” (105 genes) contained the highest number of genes (Supplementary Figure S5).

For the DEGs of the F_sex-vs-M_sex group, which represented the molecular differences between testis and ovary in 1.5Y rock breams, the GO terms with the top number of DEGs were “cellular process” (2,250 genes), “biological regulation” (1,570 genes), “membrane” (2,632 genes), “membrane part” (2,484 genes), “binding” (3,619 genes), and “catalytic activity” (2,314 genes) (Figure 1B). Although these were the same terms as the result of the F_brain-vs-M_brain group, the GO enrichment results were quite different. A total of 30 GO terms were significantly enriched, including nine development-related terms, such as “nervous system development” and “multicellular organism development” (Supplementary Figure S6). Further KEGG annotation results showed that the terms of “signal transduction” (3,077 genes) and “signaling molecules and interaction” (240 genes) were classified with the highest number of genes (Figure 2B). In addition, 40 pathways were significantly enriched, and the pathways of “human papillomavirus infection” (652 genes) and “endocytosis” (529 genes) contained the highest number of genes (Supplementary Figure S7).

A Venn diagram representing the DEGs in the F_brain-vs-M_brain and the F_sex-vs-M_sex group (Supplementary Figure S8) illustrated 335 DEGs that were specific to the F_brain-vs-M_brain group, and 17,544 DEGs specific to the F_sex-vs-M_sex group, with 1,513 overlapping. Then, we set |log2Ratio| ≥ 2 (fold change ≥ 4) as a filtering threshold to identify significant DEGs. For the DEGs specific to the F_brain-vs-M_brain group, 103 significant DEGs were identified, and the GO terms “extracellular region” and “hormone activity” were significantly enriched, which included 11 and five genes, respectively. In terms of the DEGs specific to the F_sex-vs-M_sex group, 11,904 significant DEGs were identified, and 51 GO terms (Supplementary Figure S9) and 84 KEGG (Table S3) pathways were further significantly enriched, with the pathways of “PI3K-Akt signaling pathway” (388 genes), “MAPK signaling pathway” (351 genes), “Ras signaling pathway” (289 genes), and “Rap1 signaling pathway” (288 genes) in the top ranking of highest number of DEGs. For the overlapping DEGs, 284 significant DEGs were obtained, and 13 GO terms were further significantly enriched, which mostly were development-related, including “ventral spinal cord development,” “nervous system development,” and “forebrain development” (Supplementary Figure S10).

### Validation by qPCR

**FIGURE 3:**
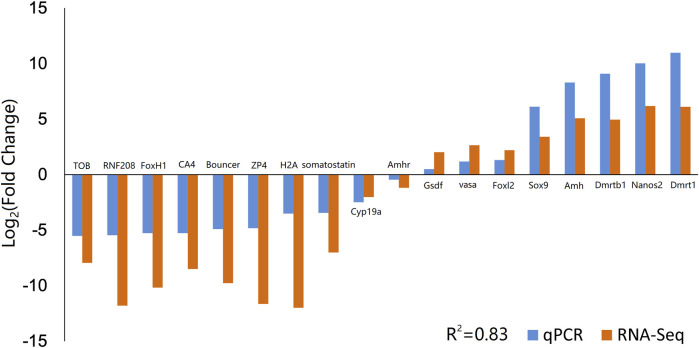
Validation of RNA-Seq data by qPCR. Comparison of the relative log_2_ (Fold Change) values of 18 genes between RNA-Seq and qPCR results. qPCR, quantitative PCR.

To validate the reliability and accuracy of the RNA-Seq results, 18 DEGs were selected for qPCR analysis, including *Amh*, *Amhr*, *Cyp19a*, *Dmrt1*, *Dmrtb1*, *Foxl2*, *Gsdf*, *Nanos2*, *Sox9*, *vasa*, *ZP4*, *Bouncer*, *CA4*, *FoxH1*, *H2A*, *RNF208*, *TOB*, and *somatostatin* (Figure 3). The coefficient of determination (*R* ^2^) between the Log_2_ (fold change) values of RNA-Seq and the qPCR results was 0.83, thus indicating good consistency.

### Tissue Distribution Analysis by qPCR

**FIGURE 4:**
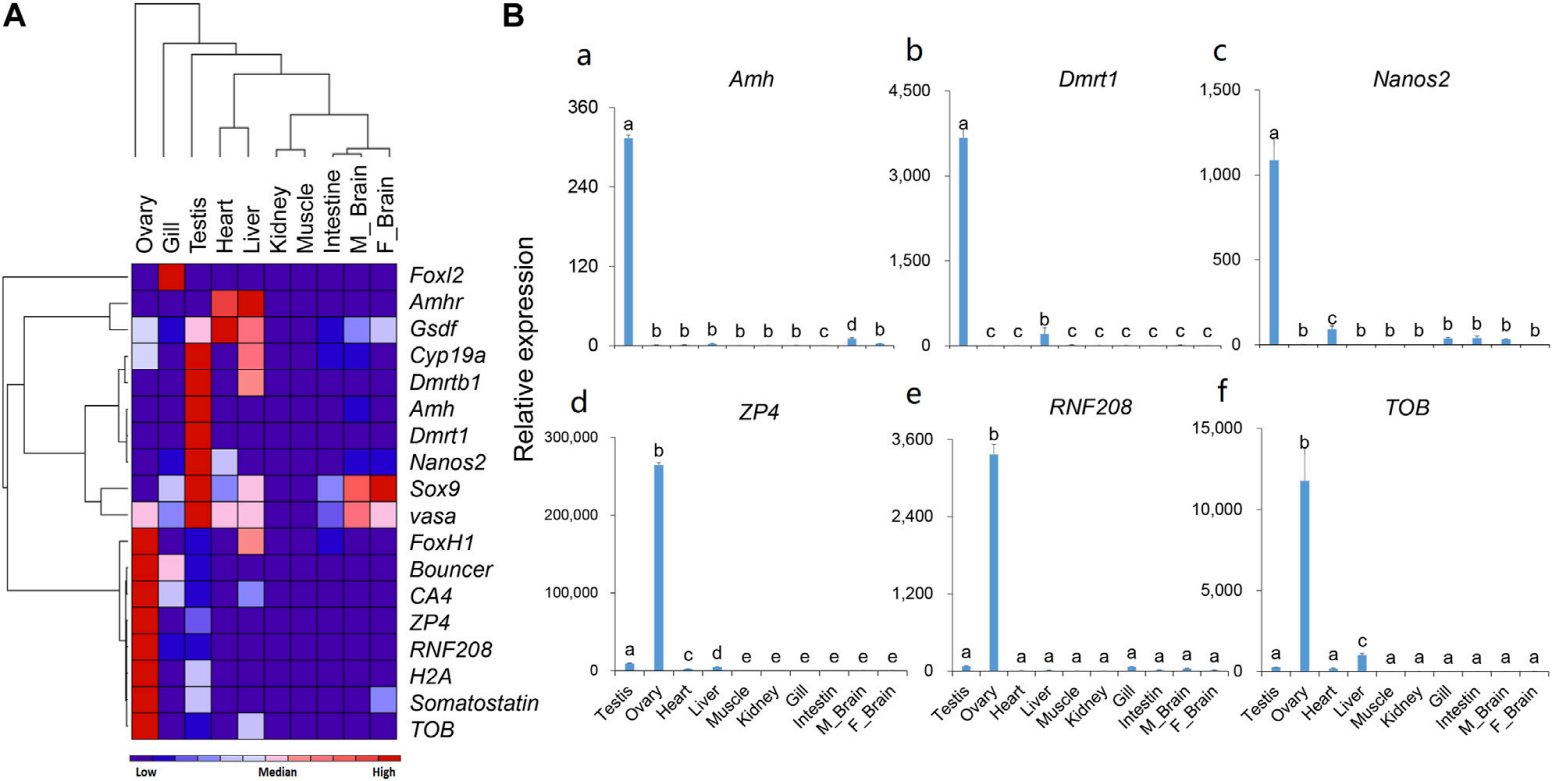
The expression patterns of selected genes in different tissues of the rock bream. **(A)** Heat map of the expression patterns for 18 selected genes; Red indicates relative high expression, blue indicates relative low expression, and color depth indicates relative expression levels; **(B)** Detailed expression patterns of selected genes (the histograms for the rest 12 genes are shown in Supplementary Figure S11).

According to the RNA-Seq results, we selected male-or female-biased expressed genes and analyzed their expression in different tissues by qPCR. The tissue distribution results (Figure 4) showed that for most of the male-biased genes, such as *Dmrt1*, *Dmrtb1*, *Amh*, *Nanos2*, and *Sox9*, their expression levels in the testis were significantly higher than in other tissues. However, we also detected high expression of *Dmrtb1* in the liver, *Sox9* in the liver and brain, and *Amhr* and *Gsdf* in the heart and liver. As for the female-biased genes, the expression levels of *ZP4*, *CA4*, *RNF208*, *H2A*, *somatostatin*, and *TOB* were significantly higher in the ovary than those in other tissues. In addition, *Bouncer* showed relatively high expression in the gills, and *FoxH1* was highly expressed in the liver. Interestingly, although the expression levels of *vasa* peaked in the testis, it was also relatively highly expressed in the ovary, heart, liver, and both male and female brains (Figure 4A).

### Sex Dimorphic Gene Expression at Different Gonadal Development Stages

**FIGURE 5:**
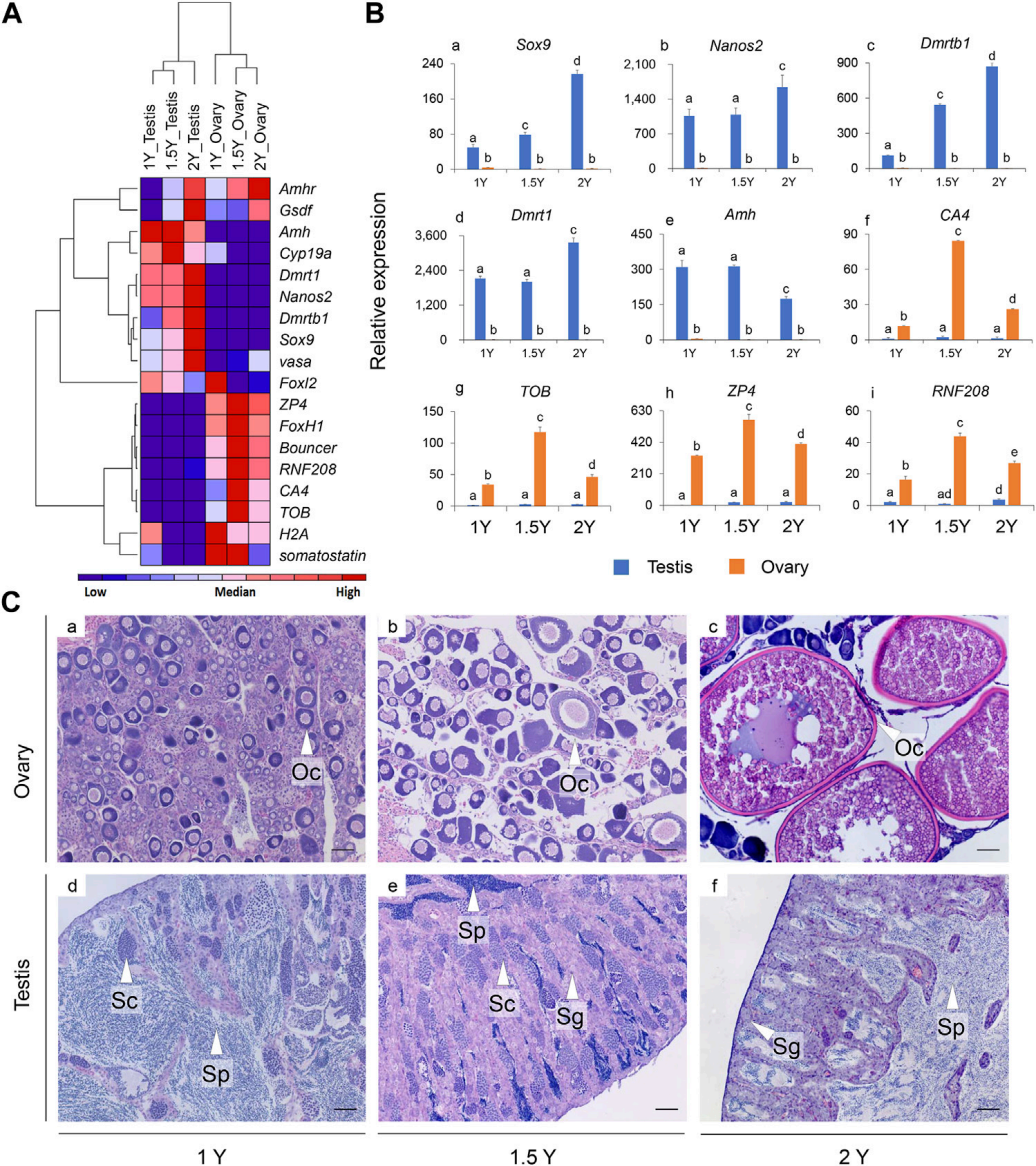
The expression patterns of selected genes in the gonads and the histological observation of the gonads for the rock breams at different development stages (1, 1.5, and 2Y). **(A)** Heat map of the expression patterns for 18 selected genes; Red indicates relative high expression, blue indicates relative low expression, and color depth indicates relative expression levels; **(B)** Detailed expression patterns of selected genes (the histograms for the rest nine genes were shown in Supplementary Figure S12). **(C)** Histological observation of the gonads at different development stages. Scale bars = 50 μm. Y, year old; Oc, oocyte; Sp, sperm; Sc, spermatocyte; Sg, spermatogonia.

The expression patterns of 18 selected genes in the gonads of rock breams at different developmental stages were analyzed by qPCR (Figure 5). We first analyzed gonadal development in rock bream using histological staining. The ovary was in stage II at 1Y, with the main cell types in the ovary being oogonias and phase I and phase II oocytes (Figures 5Ca). The testes of the rock bream at this age had developed into stage III, a large number of sperm cells were observed, and the internal structure of the testis had developed and matured (Figures 5Cd). For the 1.5Y fish, the ovary structure had developed, and mainly contained phase II–IV oocytes and emptied follicles (Figures 5Cb). In addition, a small amount of sperm was observed in the testis, along with some spermatogonia and spermatocytes (Figures 5Ce). For the 2Y fish, the ovary was in stage VI–V ovary and mainly contained phase IV and V oocytes (Figures 5Cc). At this age, the seminiferous lobules were observed to be partially melted, and the whole testis was filled with sperm cells and mature sperm, and there were still some spermatocytes (Figures 5Cf).

As shown in the heat map (Figure 5A), the expression patterns of the genes were mainly clustered into three branches: the expression levels of *Amh*, *Cyp19a*, *Dmrt1*, *Dmrtb1*, *Nanos2*, *vasa*, and *Sox9* in the testis were significantly higher than those in the ovary, and *ZP4*, *FoxH1*, *Bouncer*, *CA4*, *RNF208*, *H2A*, *somatostatin*, and *TOB* were found to be highly expressed in the ovaries, whereas *Amhr* and *Gsdf* had relatively high expression in the gonads of both 2Y male and female fish. In particular, the expression of *Dmrt1*, *Dmrtb1*, *Nanos2,* and *Sox9* increased with the age of the fish, but *Amh* showed the opposite pattern (Figure 5B). Interestingly, for most of the female biased expressed genes, such as *ZP4*, *CA4*, *RNF208*, and *TOB*, their expression in the ovaries reached peaks at 1.5Y fish instead of in 1Y or 2Y (Figure 5B).

## Discussion

In this study, for a better understanding of sex determination and differentiation in the rock bream, we investigated the gene expression profiles in the gonads and brains of males and females by conducting a comparative transcriptome analysis and screened potential sex-related genes and pathways. We detected a low number of DEGs in the brains of males and females, which indicated fewer differences in the brain between sexes at the transcriptomic level. However, many DEGs related to nervous system development were significantly enriched. In particular, 103 significant DEGs were identified to be specific in the F_brain-vs-M_brain group, and these could be potential marker genes for exploring the differences between male and female brains. The number of DEGs between the ovary and testis was quite large (19,057 DEGs), and the identified significant DEGs specific to the F_sex-vs-M_sex group was also relatively large (11,904 DEGs). In addition, many signaling pathways were further enriched, including “PI3K-Akt signaling pathway,” “MAPK signaling pathway,” “Wnt signaling pathway,” and “Janus kinase-signal transducer and activator of transcription (Jak-STAT) signaling pathway.” Many of these factors have been reported to be related to gonadal development. For example, Jak-STAT signaling pathway is an evolutionarily conserved pathway that has been reported to play a pivotal role during development in both vertebrates and invertebrates, including the development of somatic and germ line cells (Luo and Dearolf, 2001). In the present study, the expression levels of *Amh*, *Dmrt1*, *Sox9*, and *Nanos2* in testes were significantly higher than those in female ovaries and other tissues, and the expression levels of these genes in testes also varied significantly at different developmental stages, indicating that these genes might play important roles during testicular development in *O. fasciatus*.

Previous studies have reported that *Amh*, *Dmrt1*, *vasa*, *Sox9*, and *Nanos2* genes are related to sex differentiation (Schmidt et al., 2004; Daniel et al., 2005; Rodríguez-Marí et al., 2005; Alam et al., 2008; Sun et al., 2017; Zhang et al., 2018b), reproductive system development (Tsuda et al., 2003; Kuiri-Hänninen et al., 2011), and growth of aquatic species (Li et al., 2012) and are closely related to the differentiation and development of testis (Adolfi et al., 2015; Cui et al., 2017), which is consistent with our results. In addition, the transcription factor *Dmrtb1* was found to have a specific expression in testis. This gene is seldom reported in fish and currently, there are only a few reports of its occurrence in mice and humans, which showed that it might play a key role in the transition between mitosis and meiosis of mouse germ cells as well as in the process of male spermatogonial cells during meiosis (Hilbold et al., 2019). *Amh* has been studied in many vertebrates, including teleosts and mammals. Reports have shown that *Amh* is mainly expressed in Sertoli cells of the testis and granulosa cells of the ovary. It plays an important role in the process of sex reversal in medaka (*Oryzias latpes*) (Wang et al., 2018), maintaining the normal proliferation and differentiation of female and male germ cells in zebrafish (*Danio rerio*) (Zhang et al., 2020) and ensuring a balance between proliferation and differentiation. In this study, *Amh* was deteced to be significantly highly expressed in the testes of rock bream than other tissues including the ovaries, which was consistent with the results of previous studies. Although previous studies have shown that *vasa* is specifically expressed in the gonads in most teleost, where it plays an essential role in the formation of germ cells, several studies have also reported that *vasa* was expressed in tissues other than the gonads, such as the heart and brain of *O. mykiss* (Yoshizaki et al., 2000), the heart of tongue sole *Cynoglossus semilaevis* (Zhang et al., 2012). And such extragonadal expression was also detected in Mammalian (Zamboni and Upadhyay, 1983) and Amphibian (Ikenishi and Tanaka, 2000; Jia et al., 2009). Nevertheless, the functional significance of extragonadal expression of *vasa* remains to be determined. *Dmrt1* is reported to be one of the most conserved genes involved in sex determination and differentiation. Studies on Nile tilapia showed that *Dmrt1* was only expressed in mature testes, and it was also expressed in the gonads of XX tilapia after sex reversal by androgens (Wang et al., 2010). It has been reported that *Dmrt1* maintains the normal development of testes in zebrafish but does not affect the development of ovaries (Bradley et al., 2011). All these studies suggest that *Dmrt1* plays a role in testicular formation or function maintenance and can also be used as an effective molecular marker for sex identification in *O. fasciatus*. *Sox9* also appears to be involved in sex determination in many species. Its high expression was identified in the testis of Nile tilapia (Melo et al., 2019), and the testis became abnormal when *Sox9* was knocked out in male individuals. Our study showed that *Sox9* expression was significantly high in the testis and its expression increased with the maturation of the testis, suggesting that *Sox9* is closely associated with gonadal development. Although *Nanos2* has been reported as a marker gene of germ stem cells, its tissue distribution varies among species. For example, *Nanos2* was found to be specifically expressed in the male germ cells of Nile tilapia (*O. niloticus*) (Li et al., 2014; Jin et al., 2019). However, in orange-spotted grouper (*Epinephelus coioides*) and tongue sole (*C. semilaevis*), in addition to its high expression in the testis and ovaries, it was also expressed in small amounts in tissues, such as the brain and liver (Huang et al., 2017; Sun et al., 2017). In the present study, *Nanos2* was detected to be significantly highly expressed in the testis and relatively low in the heart, gill, and brain, suggesting testis-specific expression.

Some female-biased expressed genes were also investigated in this study, including *ZP4*, *Bouncer*, *RNF208*, *FoxH1,* and *TOB*. The expression levels of these genes in ovaries were significantly higher than those in testes or other tissues. Moreover, the expression levels of these genes in the ovary were also significantly different at different developmental stages, indicating that they might play important roles in female gonad development in *O. fasciatus*. Based on the information available from a limited number of previous studies, *ZP4* is mainly expressed in females in mammals, such as humans, pigs, and cows, and plays a regulatory role in sperm-egg binding, probably as a receptor or an auxiliary receptor (Meczekalski et al., 2015; Zou et al., 2019). Our results also showed a significant female-biased expression of *ZP4*. It has been reported that *Bouncer* is a key fertilization factor required for sperm-egg interaction in zebrafish (Herberg et al., 2018) and is a key determinant of species-specific fertilization in fish, which is mainly expressed in the ovary. In this study, the expression patterns of *Bouncer* were similar to those reported in previous studies, with a significantly lower expression level in the testis than in the ovary. There are few studies on this gene in fish, and no clear conclusion has been reached yet. *FoxH1* was reported as a maternally expressed gene in zebrafish (Pogoda et al., 2000; Sirotkin et al., 2000; Christopher et al., 2010), and studies in tilapia (*O. niloticus*) showed that *FoxH1* was expressed in phase I and II oocytes (Yuan et al., 2014). Our results also showed that the expression of *FoxH1* was significantly higher in the ovary than in the testis and other tissues, suggesting it might play important roles in the ovary.

It has been reported that the *Amh* signals through Amh receptors (Amhr) to regulate differentiation and the growth of target cells. In medaka (*Oryzias latipes*), *Amhr2* was detected to be expressed in somatic cells of the gonads and showed no sexually dimorphic expression during gonad development (Klüver et al., 2007). Similarly, in the spotted scat (*S. argus*), *Amhr2* was also reported to be expressed in the both male and female gonads, suggesting that it could plays an important role in the development of gonads in both male and female (Wang et al., 2018). The gene *Gsdf* is the master sex determination gene in medaka (*O. luzonensis*) (Myosho et al., 2012) and Nile tilapia (Jiang et al., 2016), where it showed male-specific high expression. However, in the olive flounder (*Paralichthys olivaceus*) (Liu et al., 2017) and the Nile tilapia (Kaneko et al., 2015), *Gsdf* was detected to be predominantly expressed in the Sertoli cells and neighboring spermatogonia in testis, but also detectable in the somatic cells surrounding the oogonia cytoplasm of oocytes. In this study, *Amhr* and *Gsdf* were also detected to have relatively high expression in the gonads of both 2Y male and female fish, which suggested that they might be involved in gonad formation and maintenance in both male and female. Overall, the qPCR results of selected genes suggested that male-or female-biased genes might be suitable molecular makers during gonadal development of the rock bream.

In conclusion, the present study characterized and identified a number of potential genes and pathways associated with sex determination and gonad development in rock bream (*O. fasciatus*) using transcriptional analysis. Several sex-biased genes were screened, including male-biased genes, such as *Dmrt1*, *Dmrtb1*, *Amh*, *Nanos2,* and *Sox9*, and female-biased genes, such as *ZP4*, *CA4*, *RNF208*, *H2A*, *somatostatin*, and *TOB*. Most of these genes showed gonad-specific (ovary or testis) expression in tissue distribution analysis, and their expression patterns during different gonadal development stages confirmed the sex dimorphic expression, indicating that they are potential molecular markers for the investigation of sex determination and differentiation in rock bream. Further studies on the function of these genes and pathways and their roles during sex determination and differentiation are needed to reveal the complex sex determination and differentiation processes of the fish with the multiple sex chromosome system.

## Data Availability

The datasets presented in this study can be found in online repositories. The names of the repository/repositories and accession number(s) can be found in the article/Supplementary Material.
