# Supplementary material for: Identification and Characterization of Dimorphic Expression of Sex-Related Genes in Rock Bream, a Fish With Multiple Sex Chromosomes
Source: Front Genet. 2021 Nov 29;12:791179. doi: 10.3389/fgene.2021.791179 (PMC8668390; doi:10.3389/fgene.2021.791179)
Supplement: Supplementary file 1 [file DataSheet1.zip › Suppl. Tables & Suppl. Figures.DOCX]

**Supplementary Materials**

**Table S1 The primers of selected genes for qPCR**

| Gene annotation | Gene abbreviation | Primers (5’-3’) |
| --- | --- | --- |
| Forkhead box L2 | *Foxl2* | -F ACAACGGCATGAGTCACCATCA |
|  |  | -R GCGGGAGCAAGCGAACTGAA |
| Doublesex and mab-3 related transcription factor 1 | *Dmrt1* | -F CAGTCAGACCTGCTGCTGGAGA |
|  |  | -R GGTGGCGTCTGGGTAGTAGGAA |
| Gonadal soma-derived factor | *Gsdf* | -F ACTATGAGGTGGGCAGCAACAG |
|  |  | -R TGGAAGGCACTGATGAGGTCTG |
| Doublesex and mab-3 related transcription factor B1 | *Dmrtb1* | -F CTGCTCAACACGCCCTGGTT |
|  |  | -R GTCTGGTGGAGGTGGGTAATGC |
| Sry-related high mobility group-box gene 9 | *Sox9* | -F ATCAGTACCTGCCGCCTCACA |
|  |  | -R CTGCTGCTGCTGCTTGGACAT |
| Anti-Müllerian hormone receptor | *Amhr* | -F GCACCAAGCAACCAGGGATTCA |
|  |  | -R TGATGAGGTACTCCGCCAGACT |
| Muellerian-inhibiting factor | *Amh* | -F ACACCGCCAACATCAACAACTG |
|  |  | -R CGCACTCCTTCGCCACCATAT |
| Nanos homolog 2 | *Nanos2* | -F AGACTGCTGGAGAGGCTGTG |
|  |  | -R CCGATAGGCTGCTGACTGAACT |
| Cytochrome P450 family 19 subfamily A | *Cyp19a* | -F AATGCTCCTCGCCGCTACTTC |
|  |  | -R GCTGCCTCTCTGTCTGGGTAAG |
| Vasa | *vasa* | -F AGCCGCAACCTGGTCAATGATG |
|  |  | -R CCTTCTTGTTCCGCCGTTCCTC |
| Histone H2A | *H2A* | -F CATCGCTCCTCGCCACATCTTC |
|  |  | -R TCCTTGGGTGCCTTGGTCTTCTTA |
| Bouncer | *Bouncer* | -F GCTGCTCTGTGACAACCTGCTT |
|  |  | -R GCTCATGGTGTAGACGGTTCCTT |
| Forkhead box protein H1 | *foxH1* | -F GAGAACACGAGCAGACCATCAGA |
|  |  | -R CTCCTCTTTGTCCCGACTCCTCT |
| [Ring finger protein 208](https://www.ncbi.nlm.nih.gov/gene/727800) | *RNF208* | -F CCTCTGTGCCGCTGGATTACTTG |
|  |  | -R CTCCTCTTCCTCTGCTGCTCCT |
| [Carbonic anhydrase 4](https://www.ncbi.nlm.nih.gov/gene/762) | *CA4* | -F TTACCGTTACCTTGGCTCCTTGAC |
|  |  | -R CACTGGCTGGATGCTTCTGTAGAC |
| Somatostatin | *somatostatin* | -F CCGCCCAGAGAGACTCCAAACT |
|  |  | -R TCCTCCTCCAGAGCCTCGTTCT |
| Transducer of ERBB2 | *TOB* | -F TGCGTCTCAGTGGTGGACAGTG |
|  |  | -R GCTGCTGCTGCTCATGCTGTT |
| Zona pellucida sperm-binding protein 4 | *ZP4* | -F TTGGTGGTGTTGTGGTGGTTGTT |
|  |  | -R CTGAGGAGGAAGAGGAGGTTGGT |

**Table S2 Summary statistics of the rock bream brains and gonads transcriptome sequencing data**

| Sample | Clean Bases(Gb) | Clean Reads (M) | Clean Reads Q20/% | Clean Reads Q30/% | Clean Reads Ratio(%) |
| --- | --- | --- | --- | --- | --- |
| F_Brain_1 | 6.54 | 43.57 | 96.81 | 88.62 | 88.77 |
| F_Brain_2 | 6.59 | 43.94 | 96.75 | 88.39 | 89.53 |
| F_Brain_3 | 6.43 | 42.88 | 96.86 | 88.53 | 90.60 |
| F_Ovary_1 | 6.39 | 42.57 | 96.17 | 86.93 | 86.75 |
| F_Ovary_2 | 6.45 | 42.99 | 96.22 | 87.04 | 87.59 |
| F_Ovary_3 | 6.57 | 43.77 | 96.65 | 88.05 | 89.18 |
| M_Brain_1 | 6.66 | 44.39 | 97.17 | 89.44 | 90.44 |
| M_Brain_2 | 6.38 | 42.56 | 96.65 | 88.08 | 89.94 |
| M_Brain_3 | 6.47 | 43.13 | 96.96 | 88.76 | 94.63 |
| M_Testis_1 | 6.48 | 43.20 | 96.78 | 88.53 | 91.27 |
| M_Testis_2 | 6.65 | 44.31 | 96.60 | 87.99 | 90.27 |
| M_Testis_3 | 6.40 | 42.68 | 96.62 | 88.03 | 90.19 |
| Average | 6.50 | 43.33 | 96.69 | 88.20 | 89.93 |


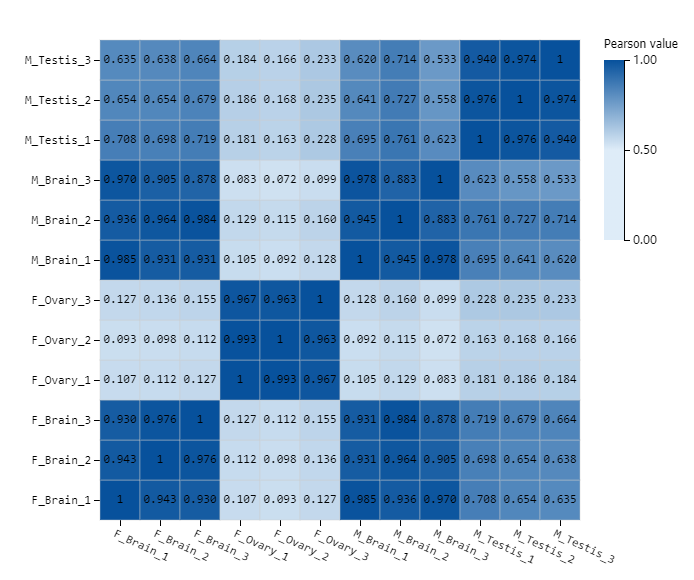


**Figure S1 The correlation coefficients between replicates of the transcriptome samples.**


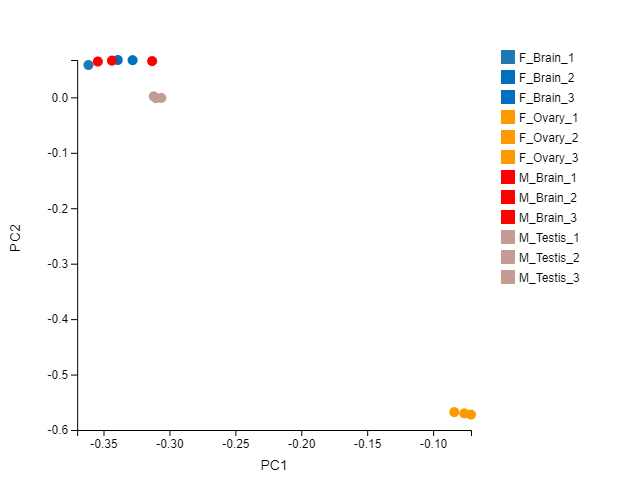


**Figure S2 Result of Principal Component Analysis (PCA) of the transcriptome samples.**


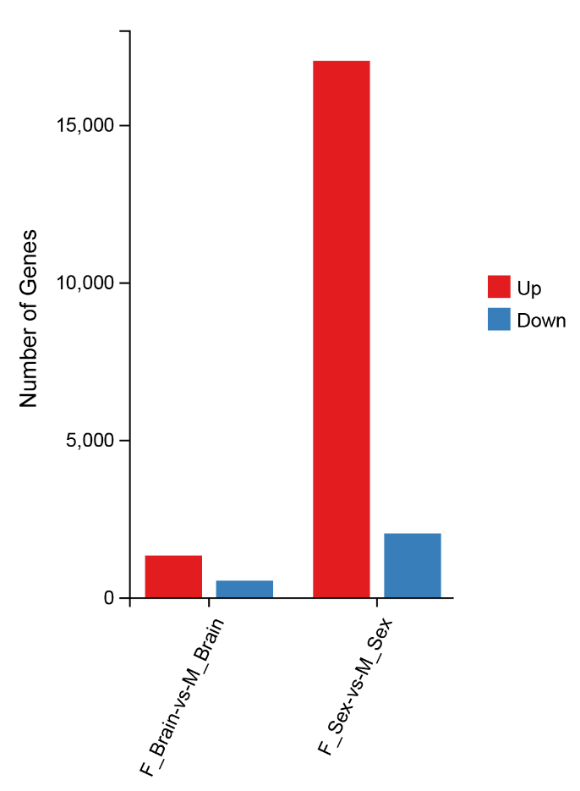


**Figure S3 Number of differentially expressed genes (DEGs) between male and female.**


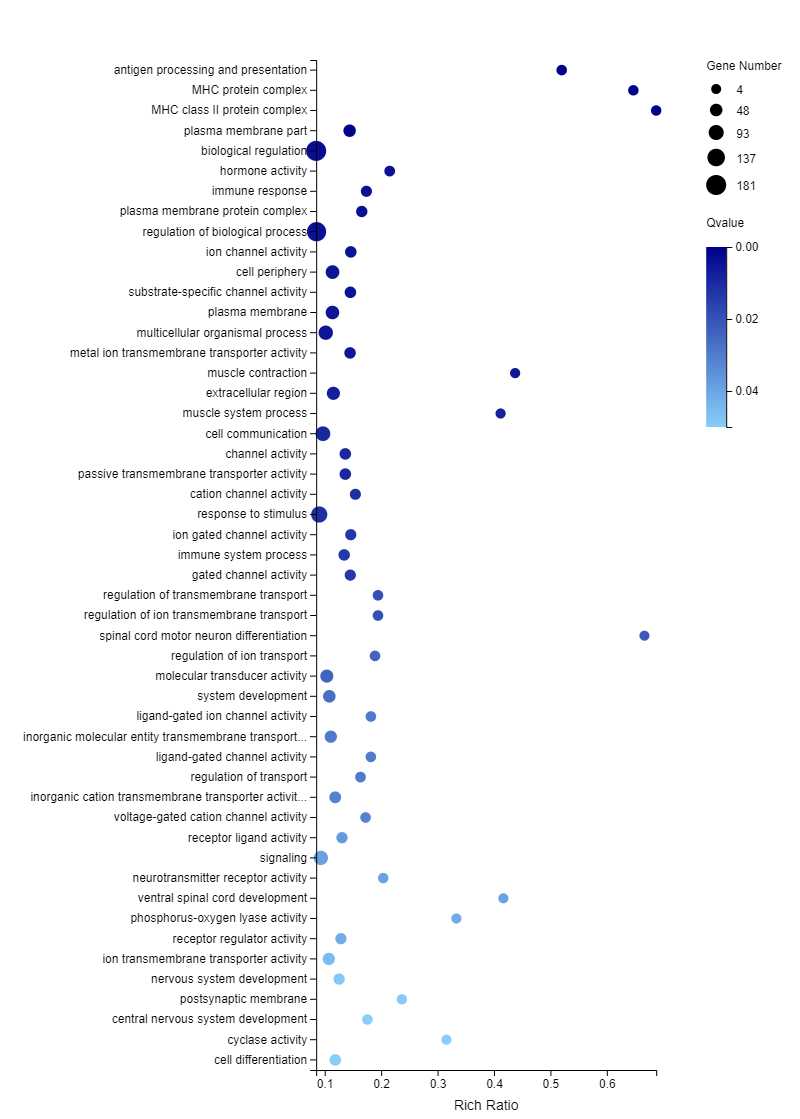


**Figure S4 GO enrichment result of F_brain-vs-M_brain group.**


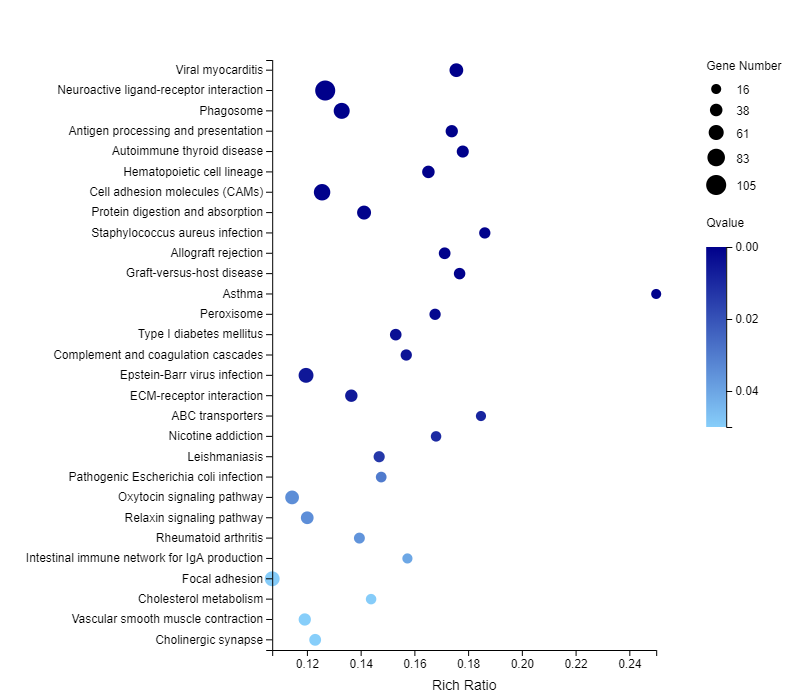


**Figure S5 KEGG enrichment result of F_brain-vs-M_brain group.**


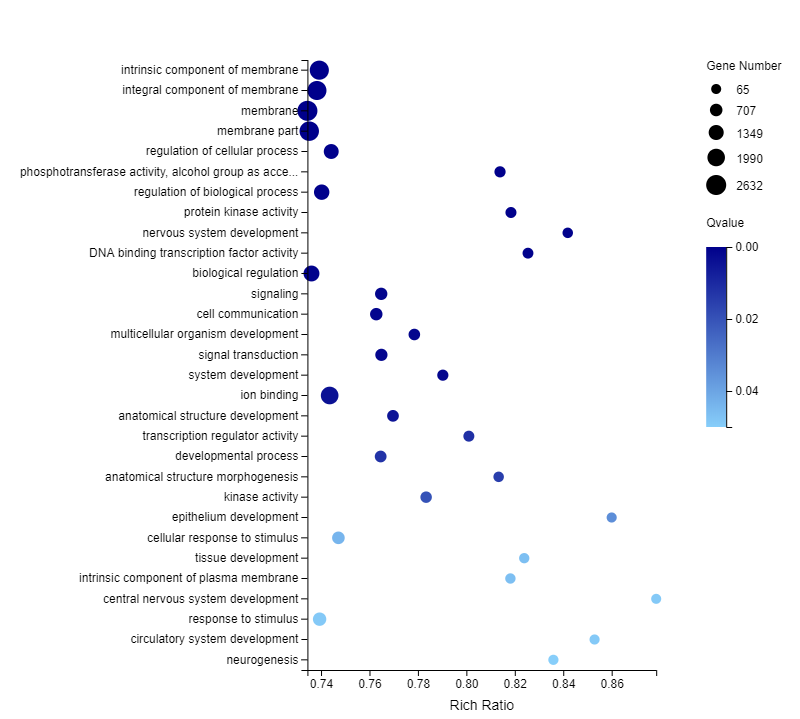


**Figure S6 GO enrichment result of F_sex-vs-M_sex group.**


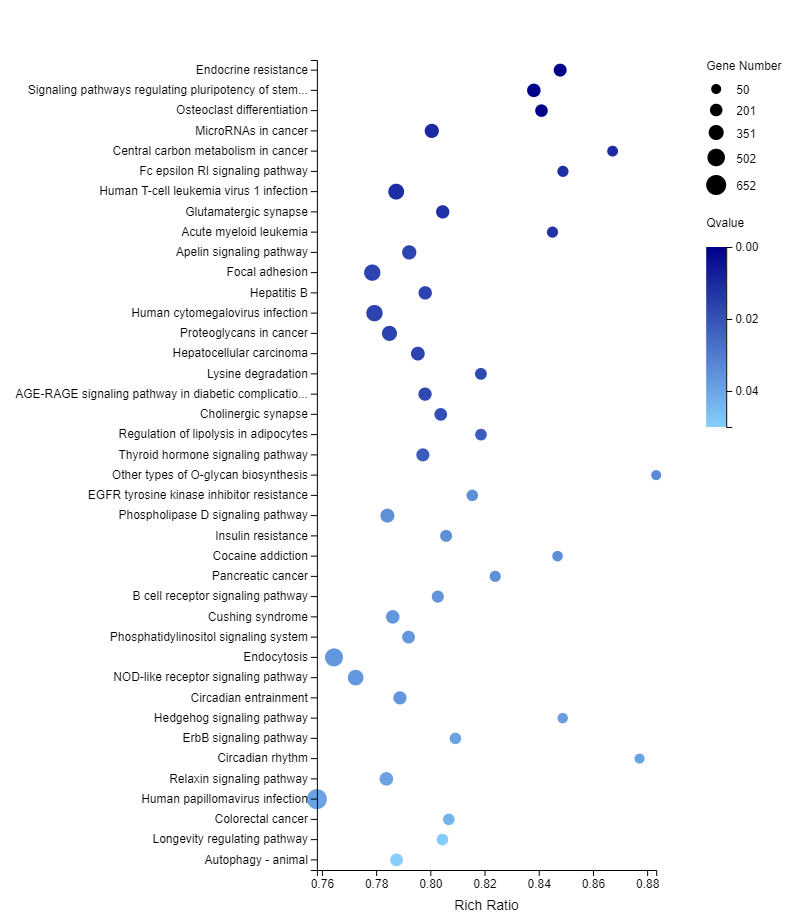


**Figure S7 KEGG enrichment result of F_sex-vs-M_sex group.**


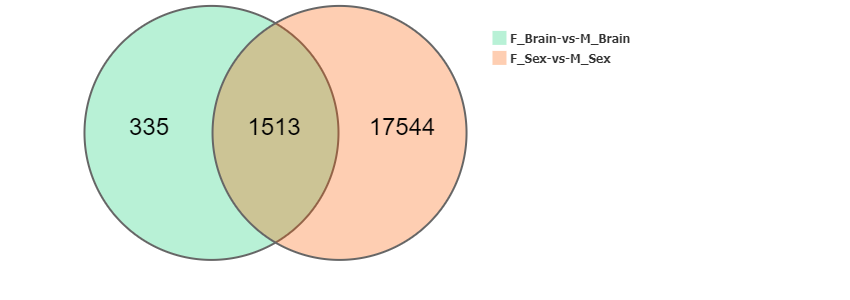


**Figure S8 The Venn diagram of the F_brain-vs-M_brain group and F_sex-vs-M_sex group.**


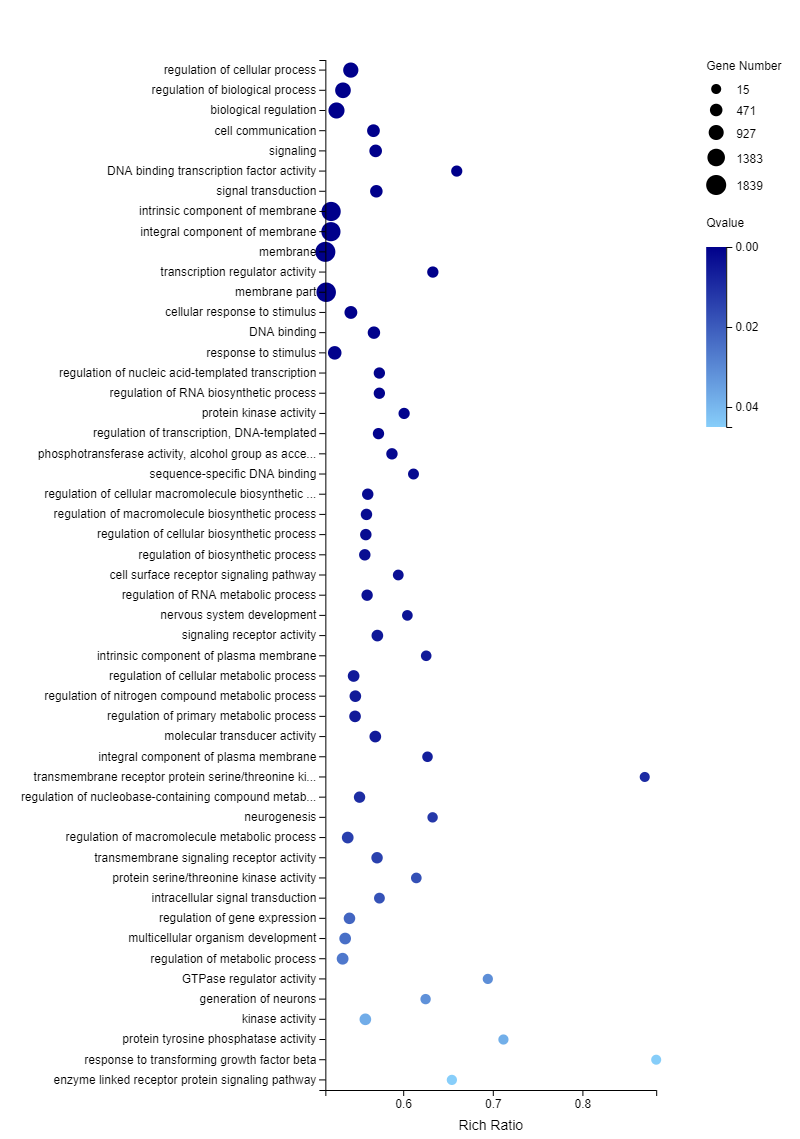


**Figure S9 GO enrichment result of significant DEGs specific to F_sex-vs-M_sex group.**


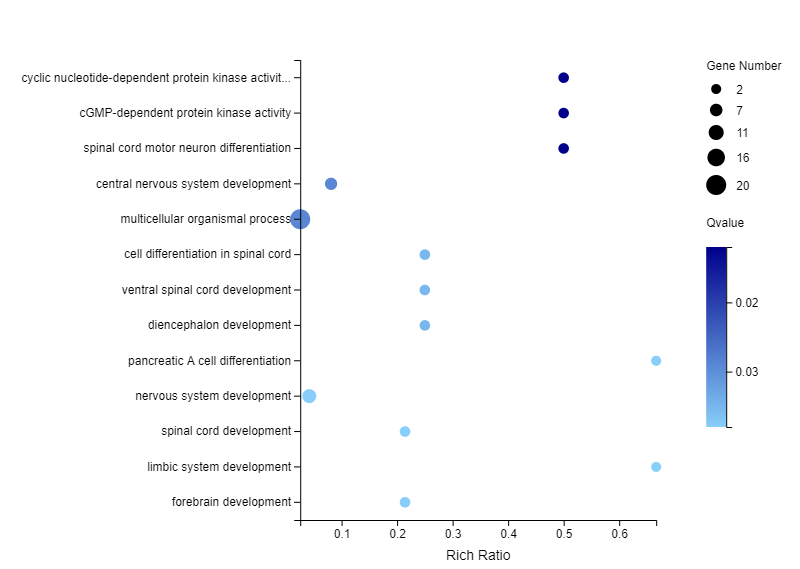


**Figure S10 GO enrichment result of significant DEGs of the overlapping of the F_brain-vs-M_brain group and F_sex-vs-M_sex group.**


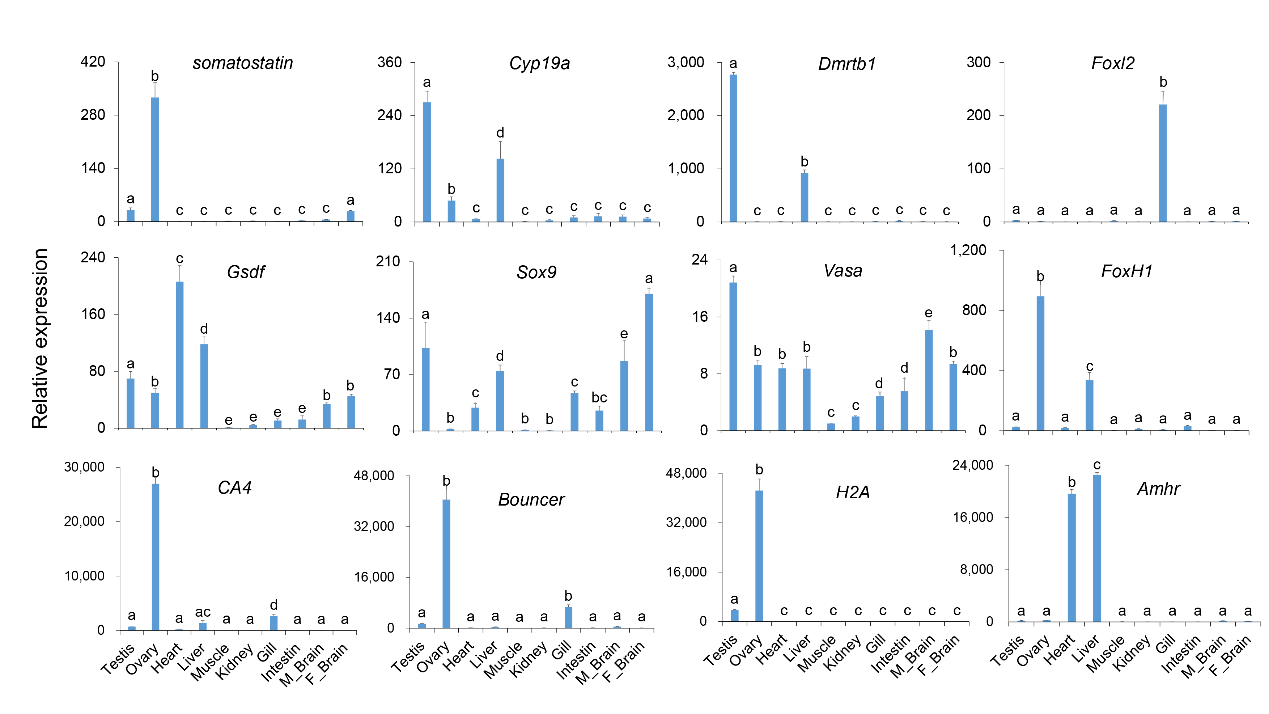


**Figure S11 Detailed expression patterns of tissue distribution for the rest 12 selected genes.**


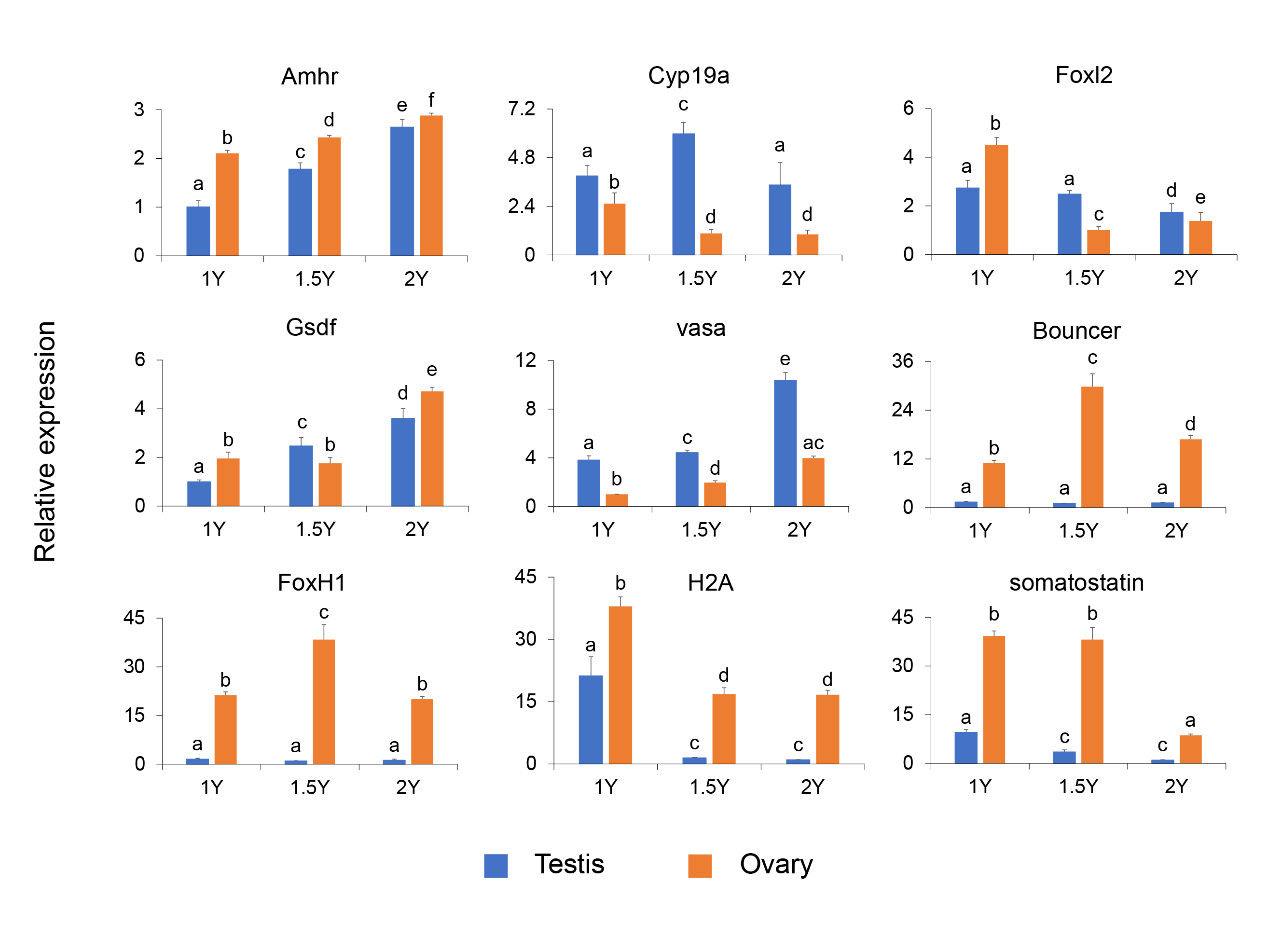


**Figure S12 Detailed expression patterns of the rest 9 selected genes in the gonads of the rock breams at different development stages (1, 1.5, 2Y).**
